# Metal work-function-dependent barrier height of Ni contacts with metal-embedded nanoparticles to 4H-SiC

**DOI:** 10.1186/1556-276X-7-75

**Published:** 2012-01-13

**Authors:** Min-Seok Kang, Jung-Joon Ahn, Kyoung-Sook Moon, Sang-Mo Koo

**Affiliations:** 1 Department of Electronic Materials Engineering, Kwangwoon University, 447-1 Wolgye-dong, Nowon-gu, Seoul, 139-701, South Korea; 2 Department of Mathematics and Information, Kyungwon Campus, Gachon University, Seongnam, 461-701, South Korea

## Abstract

Metal, typically gold [Au], nanoparticles [NPs] embedded in a capping metal contact layer onto silicon carbide [SiC] are considered to have practical applications in changing the barrier height of the original contacts. Here, we demonstrate the use of silver [Ag] NPs to effectively lower the barrier height of the electrical contacts to 4H-SiC. It has been shown that the barrier height of the fabricated SiC diode structures (Ni with embedded Ag-NPs) has significantly reduced by 0.11 eV and 0.18 eV with respect to the samples with Au-NPs and the reference samples, respectively. The experimental results have also been compared with both an analytic model based on Tung's theory and physics-based two-dimensional numerical simulations.

## Introduction

Recently, silicon carbide [SiC] has been proposed as the material of choice especially for power electronic and sensing devices operating under high temperature, fast switching, and high-power conditions mainly due to its wide bandgap (3.26 eV), high critical electric field (2.2 × 10^6 ^V/cm), superior thermal conductivity (4.9 W/Kcm), and high bulk electron mobility (900 cm^2^/Vs) of the 4H polytype [1,2]. For stable operations at high power densities and elevated temperatures, SiC diodes, including Schottky barrier diodes and junction barrier Schottky diodes, as well as SiC transistors, have been under extensive exploration with great improvements in wafer growth technology and device process.

In order to realize stable SiC devices, metal contacts to SiC with suitable physical and electrical characteristics are required. For example, Ohmic contacts with low contact resistances and Schottky contacts with controlled barrier height (*Φ*_B_) between SiC and metal are among the most important factors for determining the performance of SiC devices [3-5]. Furthermore, electrical characteristics of devices, such as voltage drop and switching speed of such devices, are dependent on the current transport behavior through the structure of the metal/4H-SiC interface. It is, therefore, of critical importance to reduce the barrier height of the metal/4H-SiC interface in order to improve the on-state voltage drop in 4H-SiC devices.

To date, extensive studies have been carried out on the properties of barrier height of various metals on n- and p-types for SiC [6,7], and many attempts have been made to modify the contact barrier height on SiC. The effect of inhomogeneities and Fermi-level pinning on Schottky contact properties has been known to be minimal, and the barrier height depends mostly on the metal work function without strong Fermi-level pinning for SiC [4,5]. Recent work on the electrical contacts to SiC includes the implementation of nanostructures such as metal nanoparticles [NPs] to modify the barrier height at metal-SiC interfaces and to alter fundamental SiC device properties by controlling the size of the metal NPs. Previous results in the literature have been primarily focused on the effect of size reduction of NPs on the characteristics of diode structures with embedded NPs, which experimentally investigates the change in transport properties of metal/semiconductor interfaces in SiC depending on the size of NPs [5-10]. However, so far, the focus has been mainly on the scaling effect of the NPs rather than on altering the electrical barrier of the NPs.

In this work, we demonstrate that the work function change in the embedded metal NPs can effectively control the barrier height change of the SiC diode structures. Our results show that incorporating NPs with a larger work function difference to the capping metal layer results in an improved barrier lowering by further enhancing the local electric field. The experimental results have also been compared with both an analytic model based on Tung's theory [11-13] and physics-based two-dimensional numerical simulations.

## Experimental details

**Figure 1:**
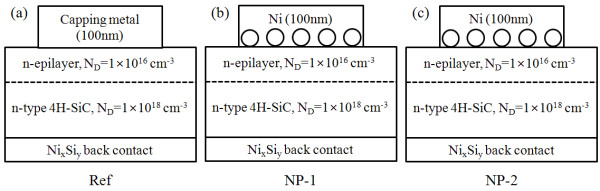
**Schematic view of Ni contacts with embedded nanoparticles on SiC**. (**a**) Ni/SiC contacts without NPs (Ref), (**b**) Ni/SiC contacts embedded with the Au-NPs (NP-1), and (**c**) Ni/SiC contacts embedded with the Ag-NPs (NP-2).

The starting materials are n-type 4H-SiC wafers with an 8-μm-thick n-type epilayer (*N*_D _= 1 × 10^16 ^cm^-3^) grown on an n+ substrate (*N*_D _= 1 × 10^19 ^cm^-3^). A large area Ohmic contact on the back was formed by e-beam evaporation of a 100-nm-thick Ni film, followed by a rapid thermal annealing process at 950°C in N_2 _for 90 s [14]. After the samples were cleaned in H_2_SO_4_:H_2_O_2 _= 4:1, the native oxide was removed by a BOE dip. A thin layer (10 nm) of metal film (Au and Ag, respectively) was then deposited on the front side of the samples by e-beam evaporation, and the samples were annealed in a quartz tube furnace at 500°C for 20 min to induce the formation and growth of the metal NPs [15,16]. As a capping layer, a 100-nm-thick Ni film was deposited on the front side of the samples to form macroscopic circular patterns with an area of 3.14 × 10^-2 ^cm^2^. We then obtained macroscopic Ni/SiC diodes with embedded NPs with different metal work function values from the capping metal/4H-SiC interface. Note that the bulk work function differences along Ni-Au and Ni-Ag are Δ*Φ*_B(Ni-Au) _which is 0.21 eV and Δ*Φ*_B(Ni-Ag) _which is 0.84 eV, respectively [17,18]. The device structures studied in this work are basically Ni/SiC contacts embedded with the metal NPs to the 4H-SiC substrate. Figure 1 shows the fabricated samples with metal NPs: Ni/SiC contacts embedded with the Au-NPs (NP-1) and Ni/SiC contacts embedded with the Ag-NPs (NP-2). Note that control samples (Ref) were also prepared for comparison by sputtering a 100-nm-thick Ni directly onto the SiC substrate without the NPs. Table 1 summarizes all the different sets of fabricated samples and process conditions.

**Table 1:** Summary of all the different sets of fabricated samples and process conditions

Sample	NPs	Capping layer	< 2*R *> (nm)	*σ* (nm)	NP Annealing
Ref	-	Ni	-	-	-
NP-1	Au	Ni	40.5	11.7	500°C, 20 min
NP-2	Ag	Ni	36.1	10.3	500°C, 20 min

The barrier height and ideality factor were compared with the physical distribution condition of the NPs as determined by field emission scanning electron microscopy [FE-SEM]. To investigate the effect of the NPs at the Ni/SiC interface on the electrical properties, current-voltage [*I-V*] and capacitance-voltage [*C-V*] characteristics of the devices were measured by using a Keithley 4200 semiconductor parameter analyzer (Keithley Instruments Inc., Cleveland, OH, USA). The experimental results have also been compared with an analytic model based on Tung's theory [11-13] and further verified by considering band diagram and electric field distribution using a physics-based two-dimensional numerical simulator Atlas (Silvaco Inc., Santa Clara, CA, USA) [19].

## Results and discussion

**Figure 2:**
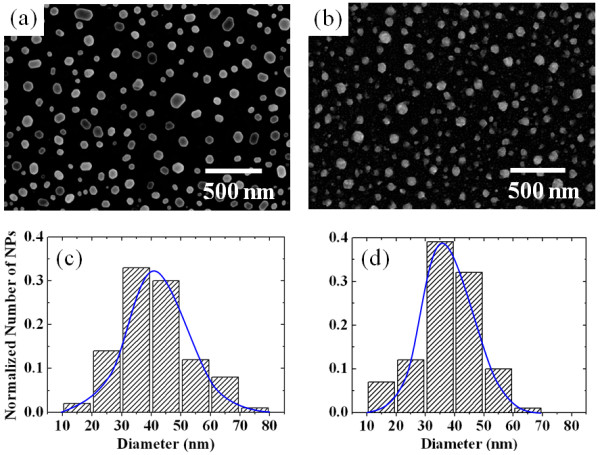
**FE-SEM surface images and distribution of relative amounts of NPs in the samples**. Representative FE-SEM images of a thin 10-nm metal film on (0001) 4H-SiC after annealing at 500°C for 20 min: (**a**) Au NPs and (**b**) Ag NPs. Distribution of the NPs' diameter in relative samples measured from the FE-SEM images: (**c**) Au NPs and (**d**) Ag NPs.

Figure 2a, b shows representative FE-SEM surface images of the nanoscale metal particles formed on SiC, where Au (NP-1) and Ag (NP-2) particles were formed after annealing 10-nm thick, corresponding metal films deposited on (0001) 4H-SiC at 500°C. It is clear that the metal (Au and Ag) films were fully agglomerated after annealing for 20 min. The physical distribution condition of the NPs has been determined by the SEM images. Figure 2c, d shows the distribution of relative amounts of the NPs in the samples sorted according to size. The diameter distribution in the samples was fitted by a Gaussian distribution and shown in a blue line in each histogram, where the peak position was taken as the average diameter (< 2*R *>), with a standard deviation [*σ*]. The average diameters of the Au and Ag NPs were 40.5 nm with a *σ *of 11.7 nm and 36.1 nm with a *σ *of 10.3 nm, respectively. It is noticeable in Figure 2 that the difference of the NPs' sizes compared to the NP-1 sample and NP-2 sample was rather small (below 6%).

**Figure 3:**
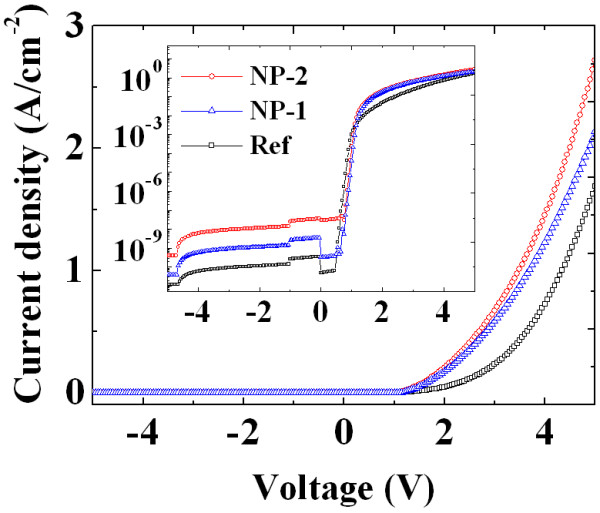
***I-V *characteristics of Ni films**. The current-voltage characteristics of Ni film without NPs (Ref), Ni film with embedded Au-NPs (NP-1), and Ni film with embedded Ag-NPs (NP-2) to n-type 4H-SiC.

Figure 3 shows the current density-voltage [*J-V*] characteristics of the as-deposited Ni contacts and samples with different embedded NPs. From *I-V *measurements, the saturation current density, effective ideality factor, and effective barrier height can be extracted in a plot of ln (*J*)-*V *characteristics. According to the thermionic emission model, the *J-V *characteristics are given by [20,21] the following equations:

(1) $$J=J_{\text{s}}\text{exp}\left(\frac{qV}{nkT}\right)\left[\text{1}-\text{exp}\left(\frac{qV}{kT}\right)\right]$$

(2) $$J_{\text{s}}=A^{*}T^{\text{2}}\;\text{exp }\left(-\frac{q\Phi_{\text{B}}}{kT}\right),$$

where *J*_s _is the saturation current density, *Φ*_B _is the effective barrier height [*Φ*_B _= *kT*/*e*ln(A*T^2^/*J*_s_)], A* is the Richard constant (for 4H-SiC, 146 A/cm^2 ^K^2^) [22], *T *is the absolute temperature, *k *is the Boltzman constant, *q *is the electron charge, and *n *is the ideality factor [*n = kT*/*e*(*dV*/*d*(ln*J*))]. The values of the effective ideality factor and barrier height were calculated from the ln (*J*) versus forward voltage *V *characteristics. Under forward voltage conditions, it clearly shows that the current value of sample NP-2 was about one order of magnitude higher than that of reference samples (10^-3 ^A/cm^2^), due to the smaller barrier height of NP-2 (0.87 eV) compared with that of Ref (1.04 eV).

**Figure 4:**
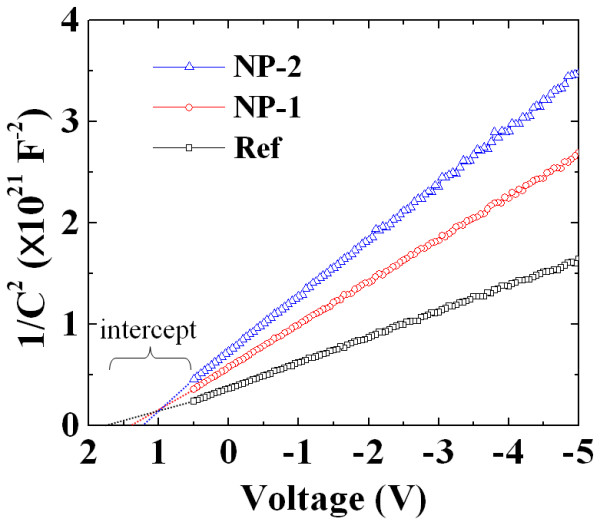
**1/*C*^2 ^versus reverse voltage characteristics**. 1/*C*^2 ^versus reverse voltage for n-type of Ni film without NPs (Ref), Ni film with embedded Au-NPs (NP-1), and Ni film with embedded Ag-NPs (NP-2) to 4H-SiC at a frequency of 1 MHz at 300 K. The contact area is 3.14 × 10^-2 ^cm^-2^.

The barrier height from *C-V *measurements was extracted as well for comparison with the *I-V *measurements. The doping concentration (*N*_D_) of the epilayers can be determined from the slope in plotting 1/*C^2 ^*versus the reverse voltage, which can be expressed as follows [23]:

(3) $${\text{N}}_{\text{D}}=\frac{2}{qK_{\text{S}}\varepsilon_{0}A^{\text{2}}\left[\frac{d\left(\text{1}∕C^{\text{2}}\right)}{dV}\right]},$$

where, *A *is the contact area of the diode (3.14 × 10^-2 ^cm^2^), *K*_S _is the semiconductor dielectric constant for 4H-SiC (6.52 at high frequency), and *ε*_0 _is the permittivity free space charge. Figure 4 shows the 1/*C*^2 ^versus reverse voltage characteristics measured at a frequency of 1 MHz at room temperature. The straight line intercepts of the 1/*C*^2^-*V *characteristics with voltage axis are obtained, and thus, the barrier height values can be given as follows [23]:

(4) $$\Phi_{\text{B}}=V_{\text{i}}+V_{\text{n}},$$

where *V*_i _is the voltage intercept, *V*_n _is the energy difference between the minimum of the conduction band and Fermi level in the bulk of n-type SiC [*V*_n _= *kT*/*e*ln(*N*_C_/*N*_D_)], and *N*_C _is the conduction band density of states for 4H-SiC at 300 K (approximately 1.66 × 10^19 ^cm^-3^) [24]. As observed from both *I-V *and *C-V *measurement results, all the samples exhibit excellent rectifying behavior with stable ideality factors.

**Figure 5:**
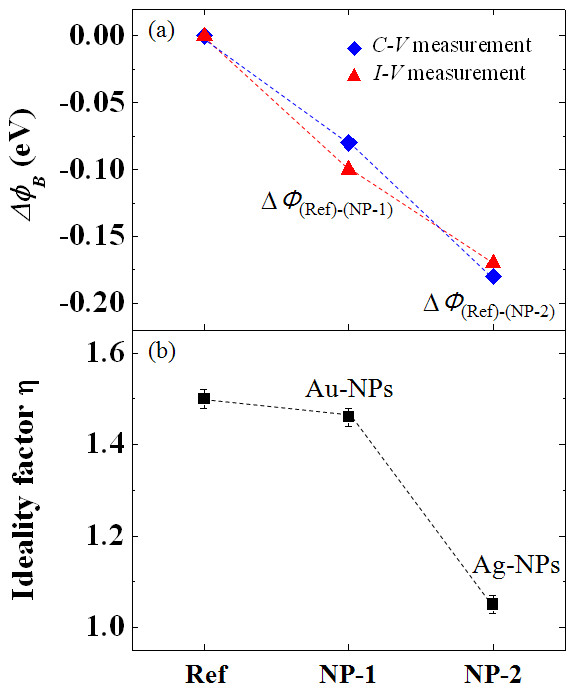
**Barrier height difference and ideality factor**. (**a**) Barrier height difference between the samples with NPs (NP-1 and NP-2) and the reference sample using *I-V *and *C-V *characteristics. (**b**) Ideality factor of fabricated diodes extracted from *I-V *characteristics.

Figure 5a shows the relative barrier height difference between the samples with NPs (NP-1 and NP-2) and the reference samples, respectively, which are extracted from *I-V *and *C-V *measurements. There is some quantitative difference between the extracted values from the two different measurements; the extracted values for the barrier heights for the reference sample and the ideality factor are *Φ*_B(*I-V*) _which is 1.04 eV and *Φ*_B(*C-V*) _which is 1.69 eV, respectively, with *n *at 1.50 for the control samples. The difference from the two different methods is commonly observed, which normally shows higher values for *C-V *measurements than those obtained from *I-V *characteristics due to additional capacitance at the interface [3,25].

The results, however, clearly suggest that the barrier height difference between the Ni/SiC contacts (Ref) and samples with embedded NPs significantly increases and that the enhancement becomes greater for Ag particles (NP-2) than for Au particles (NP-1). The values of barrier height lowering are 0.06 eV and 0.07 eV for NP-1, whereas the values are clearly increased to 0.17 eV and 0.18 eV for NP-2 as obtained from *I-V *and *C-V *measurements, respectively. Note that the reduced barrier height and improved ideality factor are attributed to the the larger difference in the metal work function of Ag than that of Au with respect to the capping metal of Ni.

In order to understand this reduction of the barrier height, we have used an analytic model by Tung [13,14], which considers the current transport theory at the metal/semiconductor interfaces with inhomogeneous barrier height [16]. In general, conventional theories of current transport, such as the thermionic emission and diffusion, are inadquate for effectively considering improved electrical behaviors associated with the NPs. The electric field *E *for the circular patch geometry of NPs at the depletion region close to the surface of the semiconductor is given by the following equation [6,13]:

(5) $$E\left(z\right)\;=V_{\text{bi}}\left(\frac{2}{w}-\frac{2z}{w^{\text{2}}}\right)-\Delta \Phi \left[\frac{1}{\sqrt{z^{\text{2}}+{R_{0}}^{2}}}-\frac{z^{\text{2}}}{\sqrt{{\left(z^{\text{2}}+{R_{0}}^{2}\right)}^{3}}}\right],$$

where *z *is the distance from the surface of the semiconductor, *w *is the depletion width, *R*_0 _is the radius of the circular patch, and Δ*Φ *is the difference of the barrier height between the capping metal and NPs.

**Figure 6:**
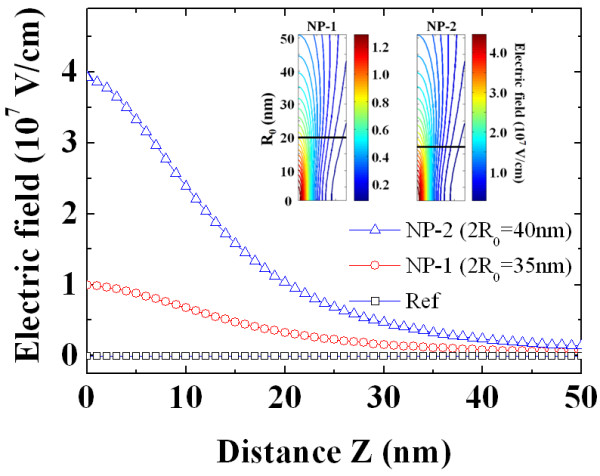
**Electric field distribution**. Comparison of the electric field distribution at the depletion region close to the surface of the 4H-SiC of different work functions using Tung's model. The inset represents the electric field distribution as a function of the size of the NPs.

Figure 6 shows the calculated electric field distribution as a function of the depth from the surface of the NPs using Equation 5. The presence of small regions with a low barrier height, *Φ*_B _- Δ, due to the difference of the barrier height between the capping metal (Ni) and NPs results in the increased electric field at the depletion region close to the surface of the semiconductor. As shown in Figure 6, the values of the electric field are estimated to be 2.6 × 10^4 ^V/cm (Ref), 0.1 × 10^7 ^V/cm (NP-1), and 3.9 × 10^7 ^V/cm (NP-2) for the given experimental conditions including the diameters of the NPs, namely, 2*R*_0 _which is 40 nm for NP-1 and 2*R*_0 _which is 35 nm for NP-2. The insets of Figure 6 show the electric field distribution as a function of the size of the NPs at n-type 4H-SiC. The electric field is increased as the small size of the NPs decreases due to the increased difference of the barrier height between Ni and the NPs. The electric field at the surface of sample NP-2 is therefore higher than that of NP-1 for a similar particle diameter.

**Figure 7:**
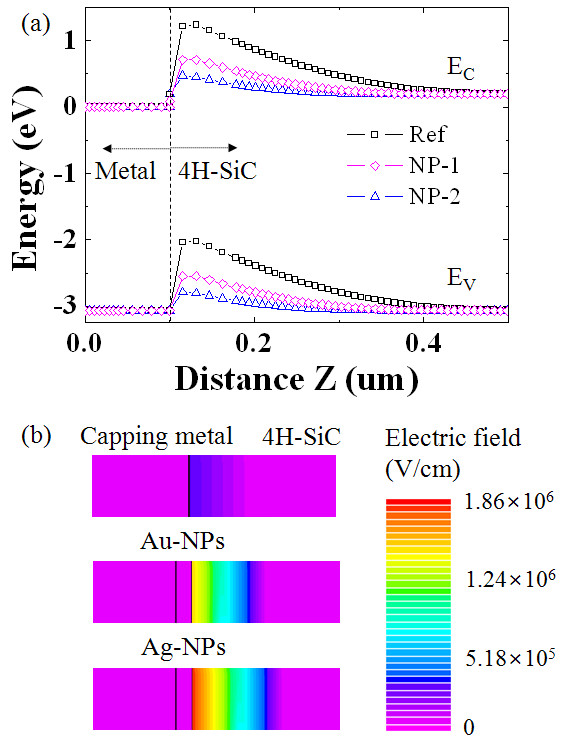
**Energy band diagram profile and electric field distribution**. (**a**) Energy band diagram profile and (**b**) electric field distribution along the cut line across the NP-substrate structures using physics-based two-dimensional numerical simulations.

To further examine this effect and understand the transport properties, we have performed two-dimensional numerical simulations. Figure 7a, b shows the electric field distribution of the metal-SiC structure, and it indicates that the maximum electric field is at the depletion region close to the surface of SiC and corresponding energy band profiles. The maximum electric field is increased up to 1.8 × 10^6 ^and 2.4 × 10^6 ^for NP-1 and NP-2, respectively, compared to the value of 5.18 × 10^5 ^for Ref. The increased electric field of the samples with the Au and Ag NPs is mainly attributed to the reduction of barrier height as the effective barrier of the conduction band at the depletion region decreases. As shown in Figure 7a, the extracted energy band diagram profiles along the cut line across the NP-substrate structures show that the reduction of barrier is more profound in NP-2 (with Ag) than in NP-1.

## Conclusions

In summary, we demostrate that the work function change in the embedded metal NPs can effectively lower the barrier height of the SiC diode structures. It has been experimentally shown that incorporating NPs (Ag) with a larger work function difference to the capping metal layer (Ni) results in an improved barrier lowering by further enhancing the local electric field. The barrier height of the fabricated SiC diode structures (NP-1; Ni with embedded Ag-NPs) has significantly reduced by 0.11 eV and 0.18 eV with respect to the samples with Au-NPs (NP-2) and the reference samples, respectively. The experimental results are in agreement with both analytic calulations based on Tung's model and physics-based two-dimensional numerical simulations, which confirm that the increased electric field of the samples with NPs is mainly attributed to the reduction of barrier height as the effective barrier of the conduction band at the depletion region of the surface decreases.

## Competing interests

The authors declare that they have no competing interests.

## Authors' contributions

MSK carried out the experiments and characterization and prepared the manuscript initially. JJA participated in the experiments on nanoparticle formation. KSM participated in the discussion of the analytical model and carried out the numerical calculation. SMK conceived the study and participated in its design and coordination.

All authors read and approved the final manuscript.

## References

[B1] Liu X Luo Z Han S Tang T Zhang D Zhou C Band engineering of carbon nanotube field-effect transistors via selected area chemical gating Appl Phys Lett 2005 86 243501 243503 10.1063/1.1944898

[B2] Guy OJ Lodzinski M Teng KS Maffeis TGG Tan M Blackwood I Dunstan PR Al-Hartony O Wilks SP Wilby T Rimmer N Lewis D Hopkins J Investigation of the 4H-SiC surface Appl Surf Sci 2008 254 8098 8105 10.1016/j.apsusc.2008.03.056

[B3] Itoh A Matsunami H Analysis of Schottky barrier heights of metal/SiC contacts and its possible application to high-voltage rectifying devices Phys Stat Sol 1997 162 389 408 10.1002/1521-396X(199707)162:1<389::AID-PSSA389>3.0.CO;2-X

[B4] Porter LM Davis RF Critical review of ohmic and rectifying contacts for silicon carbide Mater Sci Eng 1995 34 83 105 10.1016/0921-5107(95)01276-1

[B5] Sohn JI Song JO Leem DS Lee SH Nano-dot addition effect on the electrical properties of Ni contacts to p-type GaN Phys Stat Sol 2004 10 2524 2527

[B6] Lee SK Zetterling CM Östling M Åberg I Magnusson MH Deppert K Wernersson LE Samuelson L Litwin A Reduction of the Schottky barrier height on silicon carbide using Au nano-particles Solid State Electron 2002 46 1443 1440

[B7] Ruffino F Crupi I Irrera A Grimaldi MG Pd/Au/SiC nanostructured diodes for nanoelectronics: room temperature electrical properties IEEE Trans Nanotechnology 2010 9 414 421

[B8] Langhuth H Frédérick S Kaniber M Finley J Rührmair U Strong photoluminescence enhancement from colloidal quantum dot near silver nano-island films J Fluoresc 2011 21 539 543 10.1007/s10895-010-0740-z 20936331

[B9] Iucolano F Roccaforte F Giannazzo F Raineri V Temperature behavior of inhomogeneous Pt/GaN Schottky contacts J Appl Phys 2007 102 092119

[B10] Fadwa J Nilanthi W Philippe B Frédéric V Sarah YS Gilles T Michael A Pierre D Maïté CM Marie A Michel G 3D exploration of light scattering from live cells in the presence of gold nanomarkers using holographic microscopy 3D Res 2011 02 01002

[B11] Tung RT Electron transport at metal-semiconductor interfaces: general theory Phys Rev B 1992 45 13509 13523 10.1103/PhysRevB.45.13509 10001439

[B12] Tung RT Electron transport of inhomogeneous Schottky barriers Appl Phys Lett 1991 58 2821 2823 10.1063/1.104747

[B13] Sullivan JP Tung RT Pinto MR Electron transport of inhomogeneous Schottky barriers: a numerical study J Appl Phys 1991 70 7403 7424 10.1063/1.349737

[B14] Huang YP Chen CW Shen TC Huang JF Autostereoscopic 3D display with scanning multi-electrode driven liquid crystal (MeD-LC) lens 3D Res 2010 01 39 42 10.1007/3DRes.01(2010)5

[B15] Kwon JY Yoon TS Kim KB Comparison of the agglomeration behavior of Au and Cu films sputter deposited on silicon dioxide J Appl Phys 2003 93 3270 3278 10.1063/1.1556178

[B16] Spadavecchia J Prete P Lovergine N Tapfer L Rella P Au nanoparticles prepared by physical method on Si and sapphire substrates for biosensor applications J Phys Chem B 2005 109 17347 17349 10.1021/jp053194j 16853216

[B17] Clemenger K Spherical supershells in metal clusters and the transition to protocrystalline structure Phys Rev B 1991 44 12991 13001 10.1103/PhysRevB.44.12991 9999482

[B18] Chiang KC Cheng CH Jhou KY Pan HC Hsiao CN Chou CP McAlister SP Hwang HL Use of a high-work-function Ni electrode to improve the stress reliability of analog SrTiO_3 _metal-insulator-metal capacitors IEEE Trans Electron Devices 2007 28 694 696

[B19] Silvaco International Atlas User's Manual 1998 Santa Clara. CA22167625

[B20] Rhoderick EH Williams RH Metal-Semiconductor Contacts 1988 19 2 Oxford: Clarendon Press

[B21] Sze SM Physics of Semiconductor Devices 1981 2 New York: John Wiley & Sons

[B22] Pirri CF Ferrero S Scaltrito L Perrone D Guastella S Furno M Richieri G Merlin L Intrinsic 4H-SiC parameters study by temperature behaviour analysis of Schottky diodes Microelectron Eng 2006 83 86 88 10.1016/j.mee.2005.10.031

[B23] Neamen DA Semiconductor Physics and Devices 2003 3 Boston: McGraw-Hill

[B24] Bakowski M Gustafsson U Lindefelt U Simulation of SiC high power devices Phys Stat Sol 1981 162 421 440

[B25] Osvald J Numerical study of electrical transport in inhomogeneous Schottky diodes J Appl Phys 1999 85 1935 1942 10.1063/1.369185

